# Biventricular responses to exercise and their relation to cardiorespiratory fitness in pediatric pulmonary hypertension

**DOI:** 10.1152/ajpheart.00096.2024

**Published:** 2024-07-26

**Authors:** Guido E. Pieles, Dan-Mihai Dorobantu, Jessica E. Caterini, Barbara Cifra, Janette Reyes, Sara Roldan Ramos, Eilis Hannon, Craig A. Williams, Tilman Humpl, Luc Mertens, Greg D. Wells, Mark K. Friedberg

**Affiliations:** ^1^Labatt Family Heart Center, Hospital for Sick Children, University of Toronto, Toronto, Ontario, Canada; ^2^Institute of Sport, Exercise and Health, University College London, London, United Kingdom; ^3^Sports Cardiology Department, ASPETAR Orthopaedic and Sports Medicine Hospital, Doha, Qatar; ^4^Children’s Health and Exercise Research Center, University of Exeter, Exeter, United Kingdom; ^5^Congenital Heart Disease Unit, Bristol Royal Hospital for Children and Heart Institute, Bristol, United Kingdom; ^6^Faculty of Kinesiology & Physical Education, University of Toronto, Toronto, Ontario, Canada; ^7^Translational Medicine Program, Hospital for Sick Children, Toronto, Ontario, Canada; ^8^Department of Pediatrics, Hospital for Sick Children, University of Toronto, Toronto, Ontario, Canada; ^9^Department of Clinical and Biomedical Sciences, University of Exeter, Exeter, United Kingdom

**Keywords:** exercise testing, pulmonary hypertension, right ventricular function, tissue-Doppler imaging, ventricular strain

## Abstract

Despite exercise intolerance being predictive of outcomes in pulmonary arterial hypertension (PAH), its underlying cardiac mechanisms are not well described. The aim of the study was to explore the biventricular response to exercise and its associations with cardiorespiratory fitness in children with PAH. Participants underwent incremental cardiopulmonary exercise testing and simultaneous exercise echocardiography on a recumbent cycle ergometer. Linear mixed models were used to assess cardiac function variance and associations between cardiac and metabolic parameters during exercise. Eleven participants were included with a mean age of 13.4 ± 2.9 yr old. Right ventricle (RV) systolic pressure (RVsp) increased from a mean of 59 ± 25 mmHg at rest to 130 ± 40 mmHg at peak exercise (*P* < 0.001), whereas RV fractional area change (RV-FAC) and RV-free wall longitudinal strain (RVFW-S_l_) worsened (35.2 vs. 27%, *P* = 0.09 and −16.6 vs. −14.6%, *P* = 0.1, respectively). At low- and moderate-intensity exercise, RVsp was positively associated with stroke volume and O_2_ pulse (*P* < 0.1). At high-intensity exercise, RV-FAC, RVFW-S_l_, and left ventricular longitudinal strain were positively associated with oxygen uptake and O_2_ pulse (*P* < 0.1), whereas stroke volume decreased toward peak (*P* = 0.04). In children with PAH, the increase of pulmonary pressure alone does not limit peak exercise, but rather the concomitant reduced RV functional reserve, resulting in RV to pulmonary artery (RV-PA) uncoupling, worsening of interventricular interaction and LV dysfunction. A better mechanistic understanding of PAH exercise physiopathology can inform stress testing and cardiac rehabilitation in this population.

**NEW & NOTEWORTHY** In children with pulmonary arterial hypertension, there is a marked increase in pulmonary artery pressure during physical activity, but this is not the underlying mechanism that limits exercise. Instead, right ventricle-to-pulmonary artery uncoupling occurs at the transition from moderate to high-intensity exercise and correlates with lower peak oxygen uptake. This highlights the more complex underlying pathological responses and the need for multiparametric assessment of cardiac function reserve in these patients when feasible.

## Graphical abstract

**Figure:**
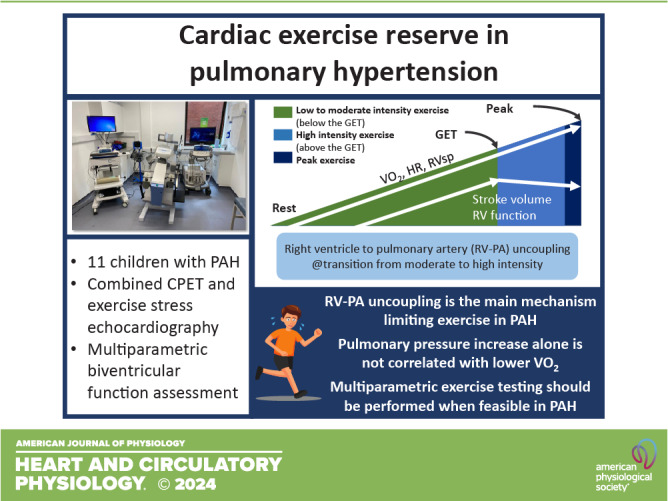

## INTRODUCTION

Pulmonary arterial hypertension (PAH) carries high morbidity and mortality, particularly in idiopathic PAH (iPAH) (1, 2). Right ventricular (RV) dysfunction and exercise capacity are major predictors of outcome, disease progression, and quality of life (3–5). In PAH, cardiopulmonary exercise testing (CPET) can elucidate the pathophysiology of exercise limitations (6) and several parameters obtained during CPET, i.e., oxygen uptake (V̇o_2_) and oxygen pulse (O_2_ pulse) correlate with clinical outcomes (7, 8).

Few studies have investigated the associations between biventricular function and exercise limitations in PAH (9), but concomitantly obtained echocardiographic and CPET data are rarely, if ever reported, especially in PAH. Importantly, a recent systematic analysis of exercise echocardiography in PAH showed that cardiac functional reserve was associated with invasive pulmonary pressure and vascular resistance measurements, peak V̇o_2_, and survival (10). Despite the findings of the review, use of exercise echocardiography remains limited, especially in children, who in general exhibit more severe PAH compared with adults (11).

Exercise echocardiography (12–14), including in children with cardiac pathology (15) and adults with PAH (16), can contribute to understanding biventricular exercise response and functional reserve, and its relationship with cardiorespiratory fitness (10). Current diagnostic tests based on exercise such as the 6-min walk test (6-MWT) and CPET (17) may help predict survival in children with iPAH (3). Nevertheless, these simple tests cannot elucidate the underlying mechanisms of RV and LV dysfunction, especially more complex aspects such as RV to pulmonary artery (RV-PA) coupling (18) or RV to LV interventricular interactions (19, 20).

We sought to describe the myocardial response to exercise and factors limiting exercise capacity in children with iPAH using concomitant CPET and exercise echocardiography. Specifically, we aimed to explore *1*) associations between peak exercise echocardiographic parameters and clinical disease characteristics, *2*) changes in right ventricular systolic pressure (RVsp) in relation to exercise intensity and responses in biventricular function, and *3*) relationships between cardiac function, RVsp, heart rate (HR), V̇o_2_, and O_2_ pulse during exercise, and how exercise intensity influences these associations.

## MATERIALS AND METHODS

### Participant Selection

Participants with a diagnosis of iPAH, based on guideline definitions at the time of recruitment (21), had >8 yr of age, and had a 6-MWT distance >400 m were prospectively recruited at the Hospital for Sick Children in Toronto. Participants were excluded if they had World Health Organization (WHO) functional class IV heart failure, musculoskeletal disease, respiratory disease (except for controlled hyperreactive airway disease), or were unable to perform exercise on a reclining bicycle. Informed written consent was obtained from participants and their guardians, respectively. After screening, 14 participants were prospectively recruited for exercise testing. Two participants subsequently withdrew, and one subject had very poor acoustic windows, precluding most measurements, resulting in 11 participants being included. The study was approved by the Research Ethics Board at the Hospital for Sick Children, Toronto (No. 100049873).

### Clinical Data Collection

Clinical information was collected from medical records, including demographics, medication use, most recent catheterization data, and spirometry data at rest. The 6-MWT was performed according to a standardized protocol (3) at least 1 h before CPET.

### Exercise Testing

An incremental CPET on a recumbent cycle ergometer (45° inclination) (eBike EL, GE Healthcare, Milwaukee, WI) following a modified Bruce protocol was performed in all participants. The test was stopped at volitional exhaustion or when unable to maintain a pedaling frequency of 60 rpm (22).

Work rates were set as follows: participants pedaled at 0 W for 3 min, with a 20-W increase in work rate every 3 min. Initially, the first step had a 20-W work rate, used for the first participant only, which proved difficult and was changed for the remainder of tests to include a 0-W step. Participants were monitored with 12-lead electrocardiogram (ECG), noninvasive blood pressure, and pulse oximetry during testing. Breath-by-breath measurements were done using a metabolic cart (METAMAX 3B, CORTEX Biophysik GmbH, Leipzig, Germany), calibrated for barometric pressure, flow volume (3-L Syringe, Hans Rudolph, Shawnee, KS), and two-point gas composition. Participants were fitted with size-appropriate, low-dead space (73–114 mL), and low-resistance oronasal mask and breathing valve assemblies (V-mask 7400 series, Hans Rudolph, Shawnee, KS). Subjective exercise intensity was assessed using a standard modified Borg scale (1–10) at the end of each stage and end of exercise. Breath-by-breath data were extracted as 10-s averages and aligned to work rate data. Within-test analysis was done using averages of the last 60 s of each exercise stage and last 30 s for peak. Gas exchange threshold (GET) (23) was calculated via piecewise regression using the V-slope method via in-house code (MATLAB, Natick) (24). All reported V̇o_2_ values are relative to body mass (mL·min^−1^·kg^−1^). The exercise intensity domains were defined based on the mean GET values for the cohort, with low- to moderate-intensity exercise being below the GET, high-intensity exercise being above the GET, and the latter including the peak intensity exercise step. These were used to aid interpretation of dynamic changes during exercise.

### Echocardiography at Rest and During Exercise

A standardized supine resting echocardiogram was performed before exercise testing using a 4- to 6-MHz transducer and E95 ultrasound system (GE HealthCare, Milwaukee) following pediatric guidelines (25). During exercise, selected views were acquired, starting 60 s into each exercise stage, then at 2 min and 6 min into recovery. In one participant, echocardiography was not performed at 2-min recovery. Images were obtained using two-dimensional (2-D), M-Mode, and spectral and tissue Doppler (TDI). LV chamber diameters were measured in the parasternal short-axis view on M-Mode images, and fractional shortening and LV eccentricity index (EccI) were calculated at end systole and end diastole. RV basal and mid diameters were measured in apical four-chamber views, and RV:LV diameter ratio was calculated using RV and LV basal measurements taken in the same LV-centered apical view at end-systole (26). Spectral pulse wave-Doppler interrogation of transmitral inflow and aortic outflow was performed from apical views. Mitral peak early (E) and late (A) diastolic waves were measured, and E/A ratio was calculated. Peak pulse-wave and TDI systolic (S′) and diastolic (E′, A′) velocities were recorded just below the level of the mitral (LV) and tricuspid (RV) valves. Fused E/A or E′/A′ waves were measured as E and E′, respectively. RVsp was estimated by the modified Bernoulli equation obtained from peak tricuspid regurgitation velocity. An assumed right atrial pressure of 5 mmHg was added to the calculated gradient across the tricuspid valve as per the clinical standard in our laboratory. As another parameter of RV function and RV-LV interaction, the RV systolic-to-diastolic (S:D) ratio was derived from RV TDI data (27, 28). Systole was defined as the interval between the end of the A′ wave and end of the S′ wave. Diastole was defined as the interval between the end of S′ and end of the A′ waves. All measurements were averaged over three consecutive cardiac cycles and are further described in Table 1.

**Table 1.** Echocardiographic parameters measured

Parameter	Description
*Right ventricular (RV) and left ventricular (LV) function*	
Tricuspid annular plane systolic excursion (TAPSE)	The distance that the RV basal plane moves toward the apex in systole measured in mm. Reflects the longitudinal component of the RV systolic function. Is influenced by passive cardiac movement during exercise.
RV-free wall peak systolic longitudinal strain (RVFW-S_l_)	Measured using speckle tracking echocardiography. It represents the percentage of deformation for the RV lateral wall from diastole to systole as an average of the three segments (basal, mid, apical). Automatically accounts for passive drift, requires good quality images.
RV fractional area change (RV-FAC)	Calculated as the percentage of change for the RV area measured on the apical four-chamber view from end-diastole to end-systole. More global measure of RV function, also including the transverse component. Affected by image quality and out-of-plane movement during exercise.
LV fractional shortening (LV FS)	Calculated as the percentage of change in LV diameter measured at the basal segment level from end-diastole to end-systole. Reflects regional basal systolic function and correlates to global function when no regional dysfunction is present.
LV peak longitudinal strain (LV-S_l_)	Measured using speckle tracking echocardiography. It represents the average percentage of deformation for the LV (apical 4 chamber view) during systole. Automatically accounts for passive drift, requires good quality images, provides information on longitudinal and global LV function.
RV and LV peak systolic and diastolic annular velocities (S′ and E′)	Measured using tissue pulsed wave Doppler imaging (TDI). Reflect the longitudinal component of the RV/LV systolic and diastolic function. Are influenced by passive movement of the myocardium and heart rate during exercise.
LV stroke volume (LV-SV)	Estimated using the subaortic time velocity integral (pulsed wave Doppler) and LV outflow tract diameter. Represents the volume of blood ejected through the aortic valve during systole. Method is influenced by angle of Doppler alignment change during exercise. Cardiac output (CO) was calculated using LV-SV and heart rate.
*RV pressure, RV-to-LV interventricular interaction*	
RV systolic pressure (RVsp)	Calculated using the velocity of the tricuspid regurgitant jet and estimated systemic venous pressure. Correlates to the pulmonary artery pressure.
LV eccentricity index (EccI)	Measure of LV chamber shape, which quantifies the degree of septal flattening at end-diastole (ED-EccI) or end-systole (ES-EccI). Calculated as the ratio between the LV diameter parallel to the interventricular septum and the LV diameter perpendicular to it. Higher EccI correlates to higher RVsp and denotes worse RV to LV interaction.
RV:LV diameter ratio	Calculated as the ratio between the basal diameter of the RV and LV in the same apical four-chamber view at end-systole. Higher values reflect a combination of RV dilation and LV compression, reflecting worse RV to LV interaction.
RV systolic-to-diastolic duration ratio (RV S:D ratio)	Represents the ratio between systolic and diastolic durations measured in this study by TDI imaging. A proposed measure of RV global dysfunction and RV to LV interaction, potentially associated with increased RVsp.

### Speckle Tracking Analysis

Two-dimensional images were obtained at 40–90 frames/s in the apical four-chamber (A4C), modified RV A4C, and parasternal basal LV short-axis views. Speckle tracking analysis was performed offline on one manually selected best cardiac cycle (ECHOPAC v. 112, GE Vingmed Ultrasound AS, Horten, Norway). After endocardial border and myocardial region of interest tracing, tracking quality was verified automatically and manually by the operator and approved if ≤2 segments/view were excluded. Averaged LV A4C peak systolic longitudinal strain (LV-S_l_), LV basal peak systolic circumferential strain (LV-S_c_), free-wall RV peak systolic longitudinal strain (RVFW-S_l_), six-segment average RV peak systolic longitudinal strain (RV-S_l_), and their respective segmental values were calculated. Intraobserver and interobserver intraclass correlation coefficients of exercise measurements from our research group were reported previously as 0.75–0.98 for strain parameters and 0.79–0.93 for TDI parameters (13, 29, 30).

### Statistical Analysis

Frequencies are reported as both counts and percentages. Continuous values are reported as means ± SD (tables) and model-estimated margins with 95% confidence intervals (CI, repeated measures longitudinal change figures). Associations between baseline characteristics and peak echocardiographic measurements were explored using a Pearson’s correlation matrix. Coefficients and *P* values were reported for each association. The following baseline/disease characteristics were considered: V̇o_2_, minute ventilation/carbon dioxide production (V̇e/V̇co_2_) slope, V̇o_2_ at the gas exchange threshold (GET), peak O_2_ pulse, baseline pulmonary vascular resistance index (PVRi), baseline invasive right ventricle systolic pressure (RVsp), 6-min walk test distance (6-MWTD), forced expiratory volume at 1 s (FEV1). The following peak exercise echocardiographic measurements were considered: left ventricular end-diastolic eccentricity index (LVED-EccI), left ventricular end-systolic eccentricity index (LVES-EccI), LV stroke volume (LV-SV), LV fractional shortening (LV-FS), lateral left ventricular mitral (LV) peak systolic velocities (LV-S′), lateral LV peak diastolic velocity (LV-E′), LV E/E′, LV peak systolic apical four chamber longitudinal strain (LV-S_l_), peak systolic LV basal circumferential strain (LV-S_c_), RVsp, tricuspid annular plane systolic excursion (TAPSE), RV fractional area change (RV-FAC), RV tricuspid peak systolic annular velocity (RV-S′), RV tricuspid peak diastolic annular velocity (RV-S′), and RV systolic-to-diastolic ratio (RV S:D).

To account for repeated measures and data missing at random within each exercise step, linear mixed models with participant cluster as random effect term (intercept) and The Kenward–Roger approximation of degrees of freedom were used to limit type 1 error inflation. The random effects models were compared with the linear model using the likelihood ratio test, and a significant random effect (intercept) for participant cluster was observed for all models (*P* < 0.05).

This methodological framework was used when analyzing *1*) the relation between cardiac parameters and RVsp, V̇o_2_, and HR; *2*) the relation between cardiac function and exercise intensity; and *3*) cardiac factors associated with V̇o_2_, O_2_ pulse, and RVsp during exercise.

For the models used in analysis 1, all data points for the repeated measures were included to allow for the relationship throughout the test to be assessed and to increase statistical power. Dependent variables used were RVsp, HR, V̇o_2_. Independent variable candidates were RV-FAC, RVFW-S_l_, RV-S′, RV-E′, TAPSE, RV S:D ratio, RV:LV diameter ratio, LVED-EccI, LVES-EccI, LV-S_l_, LV-S_c_, LV-S′, LV-E′, and LV-SV. The model was not adjusted for exercise intensity step. These relationships were also plotted using scatterplots, which showed exercise intensity steps for reference and better interpretation. To take advantage of the complete dataset, all steps between 40 W and peak (not present in all participants equally) were also included. Analyses were not adjusted for exercise intensity to allow for the relationship between dependent and independent variables to be assessed regardless of exercise intensity or timing, but the repeated-measures nature of the data (i.e., multiple observations from the same individual) was accounted for within the random effects model. Visual inspection of these scatter plots suggested some possible nonlinear relationships. These were explored using fractional polynomials by comparing the model deviance of linear and nonlinear models. Fractional polynomial models resulted in minimal, nonsignificant improvements in model fit, and only linear models were reported for simplicity.

For the models used in *analysis number 2*, the “exercise step” repeated measure variable was defined as a categorical variable, with the following strata: baseline, 0 W, 20 W, 40 W, and peak exercise (mean of 70 W), 2 min and 6 min into recovery. All measures with the number of available data points are reported at each step in the appropriate Tables. This allowed for between-stage comparisons to be performed with easy to interpret measures of effect and per-stage margins estimates to be plotted. In *analysis number 2*, dependent variables were LV and RV cardiac function parameters, and the independent variable was exercise intensity step (categorical). Changes in the dependent variables by exercise intensity were plotted using estimated model margins (and 95% confidence interval). Pairwise comparisons between exercise steps were performed, limited to comparing all exercise steps to rest and to their respective previous exercise step, with Bonferroni adjustment for multiple testing (11 tests/family).

For *analysis number 3*, on the associations between cardiac function parameters and oxygen uptake (V̇o_2_), O_2_ pulse and RVsp, all exercise step time variables were converted to percentages of the overall test length and treated as continuous longitudinal time variable. Each test had a given number of steps, 3 min in length, with resting evaluation being defined as 0%, the test end as 100%, and all in between steps related to the end-test as follows: (step number)/(total steps) × 100. This allowed for all data points to be used in this subanalysis and aligned across individuals, as well as a continuous-to-continuous interaction term to be specified in the model, reducing the required degrees of freedom and assessing the relationship changes throughout the length of exercise testing. For this analysis, the dependent variables were: RVsp, O_2_ pulse, and V̇o_2_. Independent variables were chosen from the following candidate variables: RVsp, RV-FAC, TAPSE, RV-S′, RVFW-S_l_, RV-S_l_, RV S:D, LV-FS, LV-SV, LV-S′, LV-E′, LV-S_l_, and LV-S_c_, adjusted by exercise test time, as a proportion from total exercise test length, treated as a continuous longitudinal time variable. Associations between the dependent and independent variables across length of the test were explored using a univariable longitudinal data analysis approach, including a continuous-to-continuous interaction term between the candidate-independent variables of cardiac function and the time variable. The main effect of the cardiac function-independent variable and the interaction effect was reported. To allow for appropriate interpretation of the main effect of exercise intensity, the time variable was centered around the 50% value (corresponding to the mean), and thus the main effect represents the effect at midtest. The cardiac function-independent variables were not centered on mean, as the main effect of the time variable was not of interest in this analysis. The slope of the association of interest was estimated in 10% increments across the time variable (percentage of total test length), and the time point where the association became statistically significant (*P* < 0.1 chosen) was reported. These numerical data were interpreted and summarized in a corresponding graphical abstract.

All statistical analyses were performed in SPSS v. 22.0 (IBM, Armonk, New York, NY) and STATA SE/17 (StataCorp, College Station, TX).

## RESULTS

A total of *n* = 11 participants (64%, *n* = 7 girls), with moderate to severe iPAH (mean PVRi 13.6 ± 8.9 mmHg·L^−1^·min^−1^) were included. The mean age was13.4 ± 2.9 [range, 9–17] yr, and the mean time from diagnosis was 92 ± 61 mo. The baseline characteristics are described in Table 2. Of all participants, four were on pulmonary vasodilator monotherapy, six on combination therapy, and one was without medications. Eight were classified as WHO functional class I and 3 as WHO functional class II. The cohort had significantly reduced exercise tolerance compared with available normal values in children (13), with a mean peak V̇o_2_ of 19.7 ± 5.6 mL·kg^−1^·min^−1^ (45 ± 12.2% predicted). Resting echocardiographic measurements are shown in Supplemental Tables S1–S3.

**Table 2.** Children with pulmonary arterial hypertension: demographic, anthropometric, biological, and clinical data

	Means ± SD	*n* With Available Data
Age, yr	13.4 ± 2.9	11
Time from diagnosis, mo	91.7 ± 61.1	10
Height, cm	157.2 ± 16.6	11
Body mass, kg	52.7 ± 19.3	11
BSA, kg·m^−2^	1.5 ± 0.3	11
6-MWTD, m	528.7 ± 73.3	10
NT-proBNP, pmol·L^−1^	21.3 ± 15.8	9
SBP, mmHg	103.9 ± 8.7	10
DBP, mmHg	59.9 ± 8.1	10
$\text{S}{\text{a}}_{{\text{O}}_{2}}$ at rest, %	97 ± 2.5	11
Spirometry		
FEV1, %predicted	84.9 ± 11.8	9
MVV, % predicted	76.1 ± 24	8
Cardiopulmonary exercise testing		
Peak work rate, W	67 ± 16	11
Peak V̇o_2_, mL·kg^−1^·min^−1^	19.7 ± 5.6	11
Peak V̇o_2_, % predicted	45 ± 12.2	11
$\text{S}{\text{a}}_{{\text{O}}_{2}}$ at peak exercise, %	92.1 ± 7	10
HR at peak exercise, beats/min	154 ± 23	11
Peak exercise RER	1.13 ± 0.15	10
Peak RPE, Borg units	8 ± 2	7
V̇e/V̇co_2_ slope	35.8 ± 6.5	11
Most recent cardiac catheterization		
Months from study date	27 ± 33	9
RVsp, mmHg	65.9 ± 22.8	9
mPAP, mmHg	44.3 ± 15	11
PVRi, mmHg·L^−1^·min^−1^	13.6 ± 8.9	11

Values are means ± SD; *n* = 11 children. 6-MWTD, 6-min walk test distance; BSA, body surface area; DBP, diastolic blood pressure; FEV1, forced expiratory volume in the 1st s; HR, heart rate; mPAP, mean pulmonary arterial pressure; MVV, maximum voluntary ventilation; NT-proBNP, NH_2_-terminal probrain natriuretic peptide; PVRi, pulmonary vascular resistance index; RER, respiratory exchange ratio; RPE, rate of perceived exertion; RVsp, right ventricle systolic pressure; $\text{S}{\text{a}}_{{\text{O}}_{2}}$, oxygen saturation; V̇co_2_, carbon dioxide production; V̇e, ventilatory equivalent; V̇o_2_, oxygen uptake.

Associations between peak exercise echocardiographic parameters and commonly reported resting clinical disease characteristics were explored, with the following being found: peak LV-S_l_ was associated with V̇e/V̇co_2_ slope (b = −0.9, *P* = 0.02), invasively measured PVRi (b = 0.9, *P* = 0.05) and RVsp (b = 0.9, *P* = 0.04). Peak exercise RVsp correlated to invasively measured PVRi (b = 0.8, *P* = 0.02) and RVsp at baseline (b = 0.8, *P* = 0.03). Peak RV-S′ correlated to peak O_2_ pulse (b = 0.6, *P* = 0.04). The time between the study visit and the most recent invasive catheterization varied significantly, from the same day to 7.8 years. All tested associations are detailed in Supplemental Table S4 and only include associations with >5 data points.

### Biventricular Functional Response and RV-LV Interactions during Exercise

Summary statistics of echocardiographic parameters at rest and during exercise are detailed in Supplemental Table S1 (RV measurements), Supplemental Table S2 (LV measurements), and Supplemental Table S3 (RV and LV strain).

### RV Function and Pressure Response to Exercise

**Figure 1.**
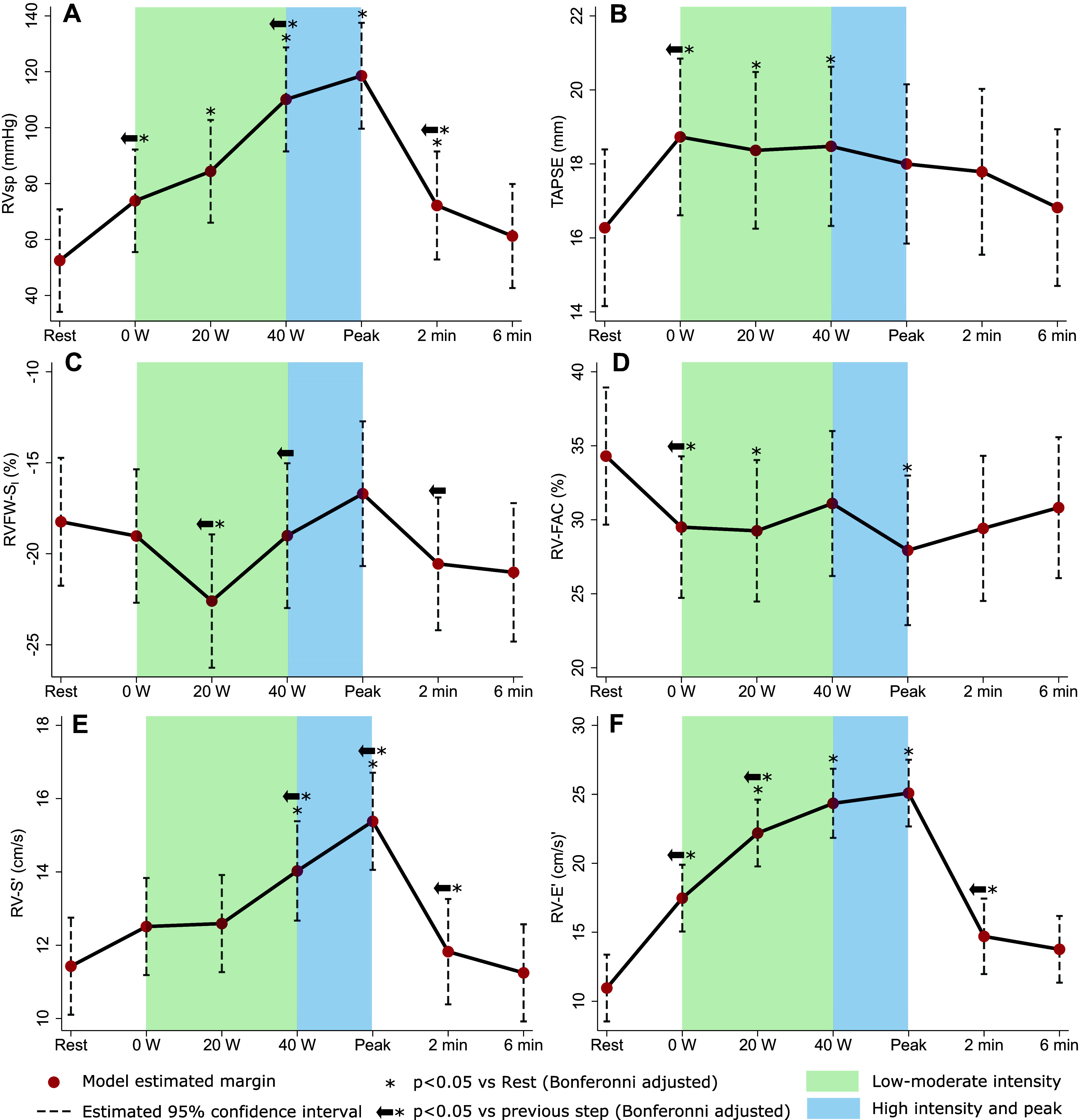
Right ventricle (RV) function and pressure response to exercise. *A*: RV systolic pressure (RVsp). *B*: tricuspid annular plane systolic excursion (TAPSE). *C*: RV free-wall (FW) peak longitudinal strain (S_l_). *D*: RV fractional area change (FAC). *E*: RV peak S′ velocity. *F*: RV peak E′ velocity. All figures show linear mixed model (exercise step as fixed effect, participant cluster as random effect) margin estimates with 95% confidence intervals.

RVsp increased continuously and markedly from rest to peak exercise (by a mean of 140 ± 66%), returning to baseline during recovery (combined *P* < 0.001; Fig. 1*A*). RV function response during exercise varied depending on the parameter measured. There was a modest increase in TAPSE (combined *P* = 0.03; Fig. 1*B*) and RVFW-S_l_, which worsened at peak exercise relative to the previous step (combined *P* = 0.05; Fig. 1*C*). RV-FAC decreased during exercise to a stable low value, returning to baseline in recovery (combined *P* = 0.09; Fig. 1*D*). In contrast, RV-S′ (combined *P* < 0.001; Fig. 1*E*) and RV-E′ (combined *P* < 0.001; Fig. 1*F*) increased with exercise, returning to baseline during recovery. Detailed results are shown in Supplemental Table S1 (“conventional” RV function parameters) and Supplemental Table S3 (RV strain parameters).

### RV-LV Interactions During Exercise

#### Biventricular size.

**Figure 2.**
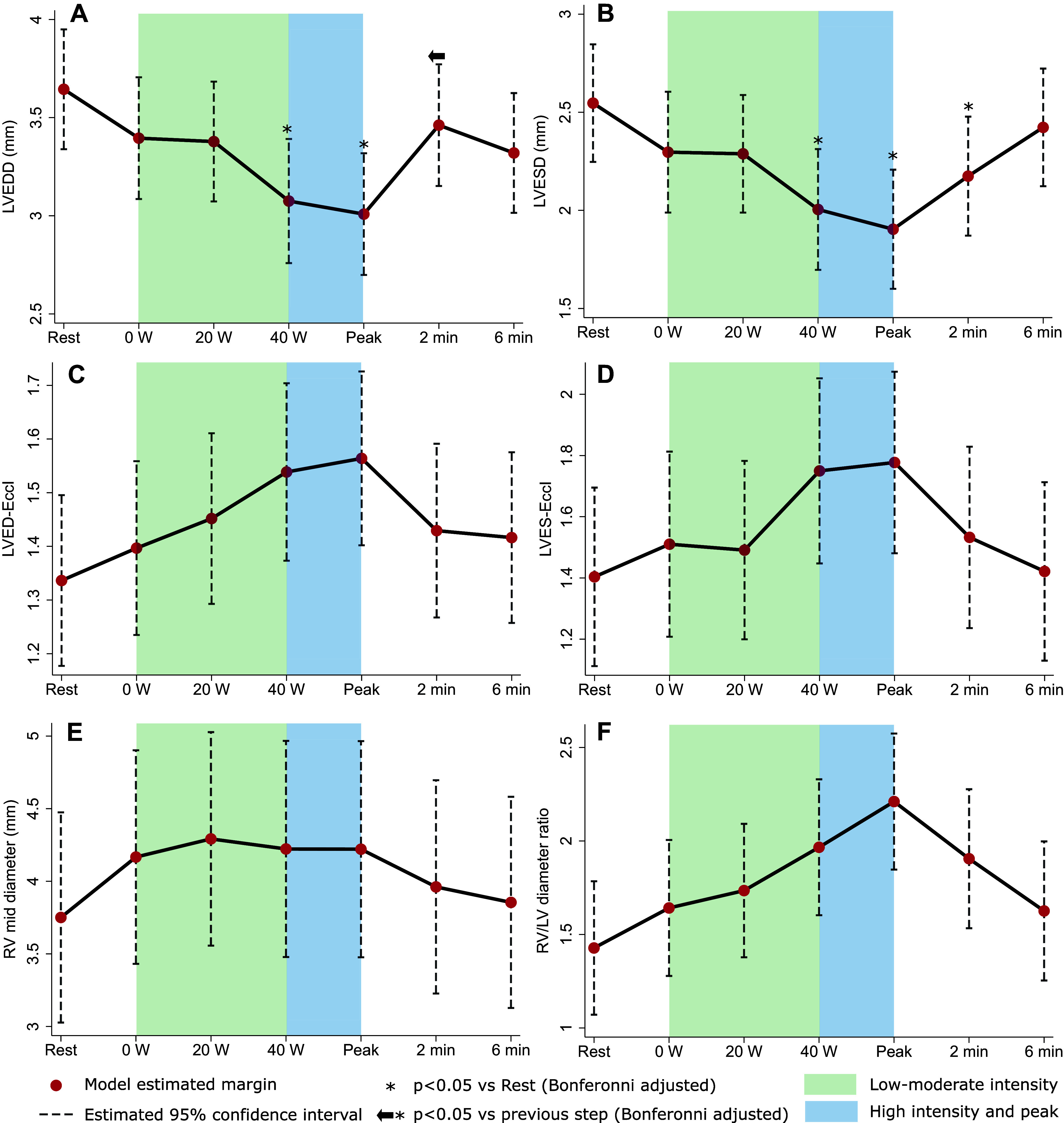
Left ventricle (LV) and right ventricle (RV) size changes in response to exercise. *A*: LV end-diastolic diameter (LVEDD). *B*: LV end-systolic diameter (LVESD). *C*: LV end-diastolic eccentricity index (LVED-EccI). *D*: LV end-systolic eccentricity index (LVES-EccI). *E*: RV middiameter. *F*: RV:LV diameter ratio. All figures show linear mixed model (exercise step as fixed effect, participant cluster as random effect) margin estimates with 95% confidence intervals.

Exercise was associated with a reduction in LV end-diastolic (combined *P* < 0.001; Fig. 2*A* and Supplemental Table S2) and end-systolic (combined *P* < 0.001; Fig. 2*B* and Supplemental Table S2) diameter, and an increase in LVED-EccI (combined *P* = 0.02; Fig. 2*C* and Supplemental Table S2) and LVES-EccI (combined *P* = 0.01; Fig. 2*D* and Supplemental Table S2). This quantitative assessment correlated with visual assessment of the interventricular septal shape during exercise. In six participants, the septum shifted from a flat septal configuration to septal bowing into the LV. In one participant, the septal configuration changed from normal to flat, in one it remained bowed into the LV throughout the test, and in three it remained flat throughout the exercise test. There was a trend for RV middiameter increase during exercise (combined *P* = 0.09; Fig. 2*E* and Supplemental Table S1), leading to a concomitant increase in RV:LV diameter ratio (combined *P* < 0.001; Fig. 2*F* and Supplemental Table S1).

#### LV function response.

**Figure 3.**
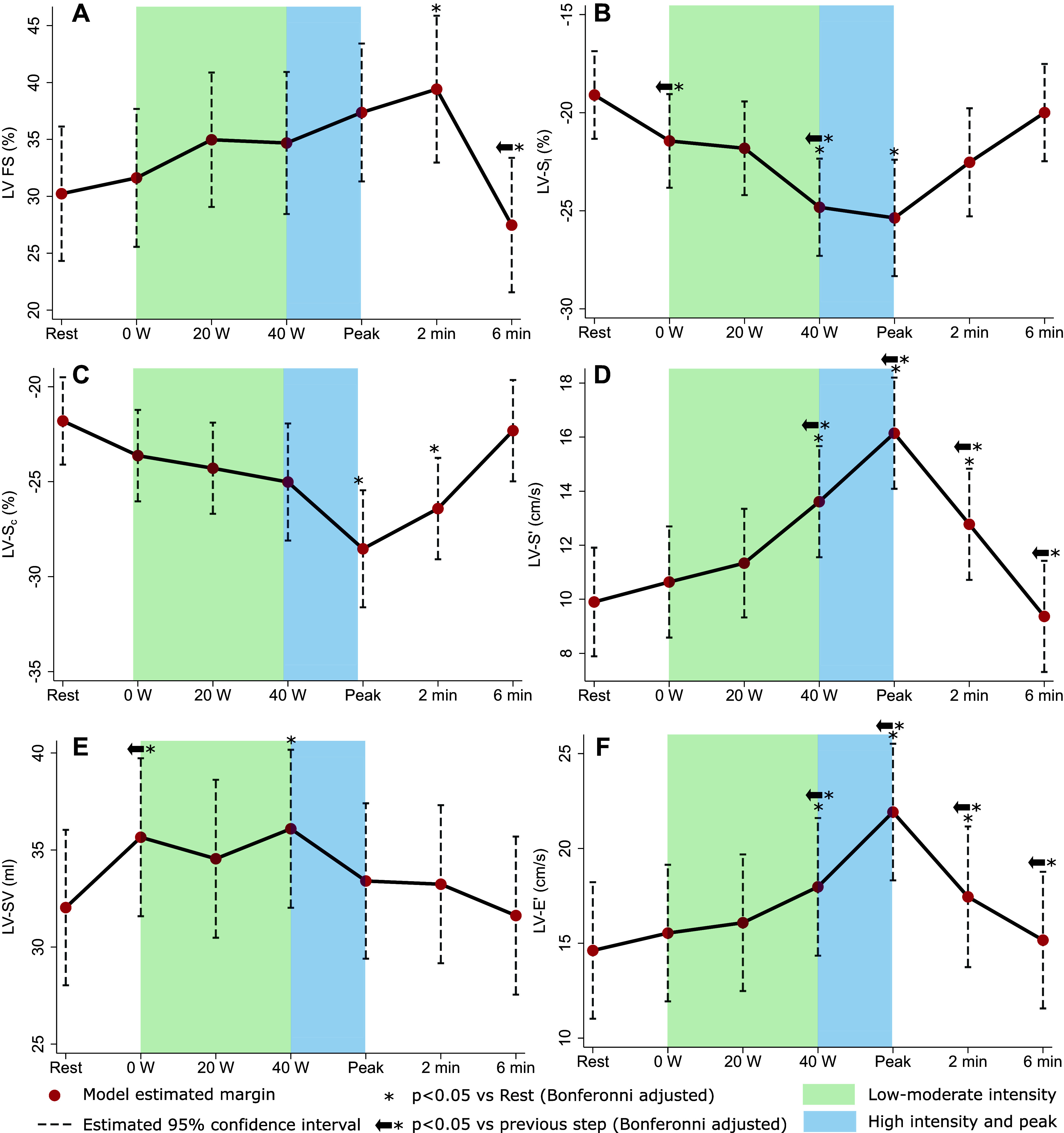
Left ventricle (LV) function response to exercise. *A*: LV fractional shortening (FS) *B*: LV apical 4-chamber peak longitudinal strain (A4C S_l_). *C*: LV basal peak circumferential strain (S_c_). *D*: LV peak lateral S′. *E*: LV lateral E′. *F*: LV E/E′. All figures show linear mixed model (exercise step as fixed effect, participant cluster as random effect) margin estimates with 95% confidence intervals.

In contrast to the RV response, LV function increased during exercise, returning to baseline in recovery as represented by LV FS (combined *P* < 0.001; Fig. 3*A*), LV-S_l_ (combined *P* < 0.001; Fig. 3*B*), LV-S_c_ (combined *P* < 0.001; Fig. 3*C*), and LV-S′ (combined *P* < 0.001; Fig. 3*D*). LV-SV increased modestly up to 40 W, then decreased to peak exercise (combined *P* = 0.04; Fig. 3*E*). There was an increase in LV-E′ (combined *P* < 0.001, Fig. 3*F*), but the E/E′ ratio remained constant throughout exercise and recovery (Supplemental Table S2). Further details and measurements are shown in Supplemental Table S2 (“conventional LV function parameters”) and Supplemental Table S3 (“LV strain parameters”).

### Biventricular Function Relation to RVsp and HR During Exercise

**Figure 4.**
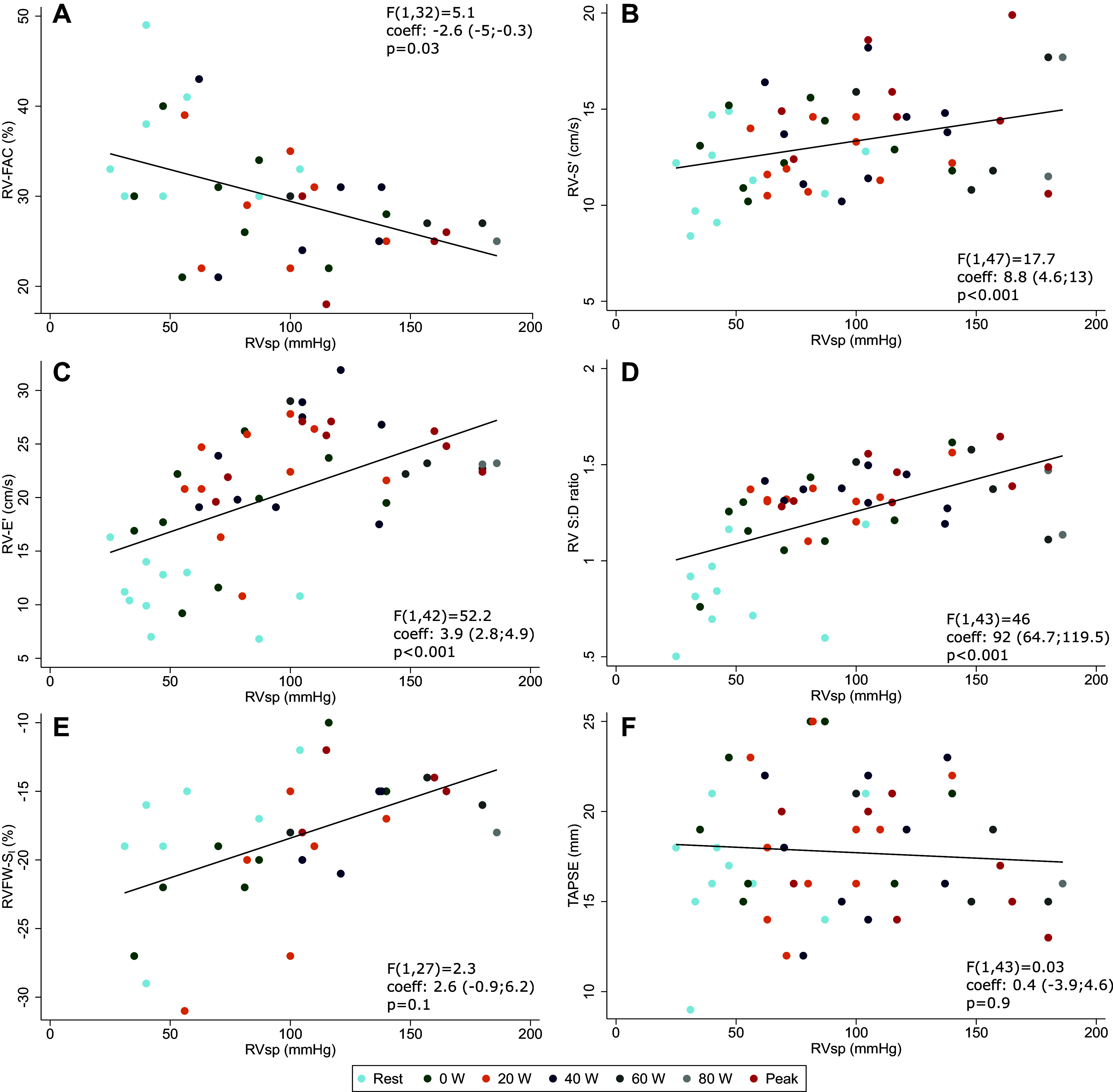
Right ventricle (RV) systolic pressure (RVsp) associations with RV systolic and diastolic function parameters throughout exercise. *A*: RV fractional area change (RV-FAC). *B*: RV peak systolic longitudinal velocity (RV-S′). *C*: RV peak longitudinal diastolic velocity (RV-E′). *D*: RV systolic-to-diastolic (S:D) ratio. *E*: RV free-wall peak systolic longitudinal strain (RV-FW S_l_). *F*: tricuspid annular plane systolic excursion (TAPSE). *F* values, coefficient, and *P* values are from mixed models with RVsp as dependent variable, cardiac function parameters as independent variable (fixed effect term), and participant cluster as random effect term, not adjusted by exercise intensity step (Table 3 and Supplemental Table S5).

**Figure 5.**
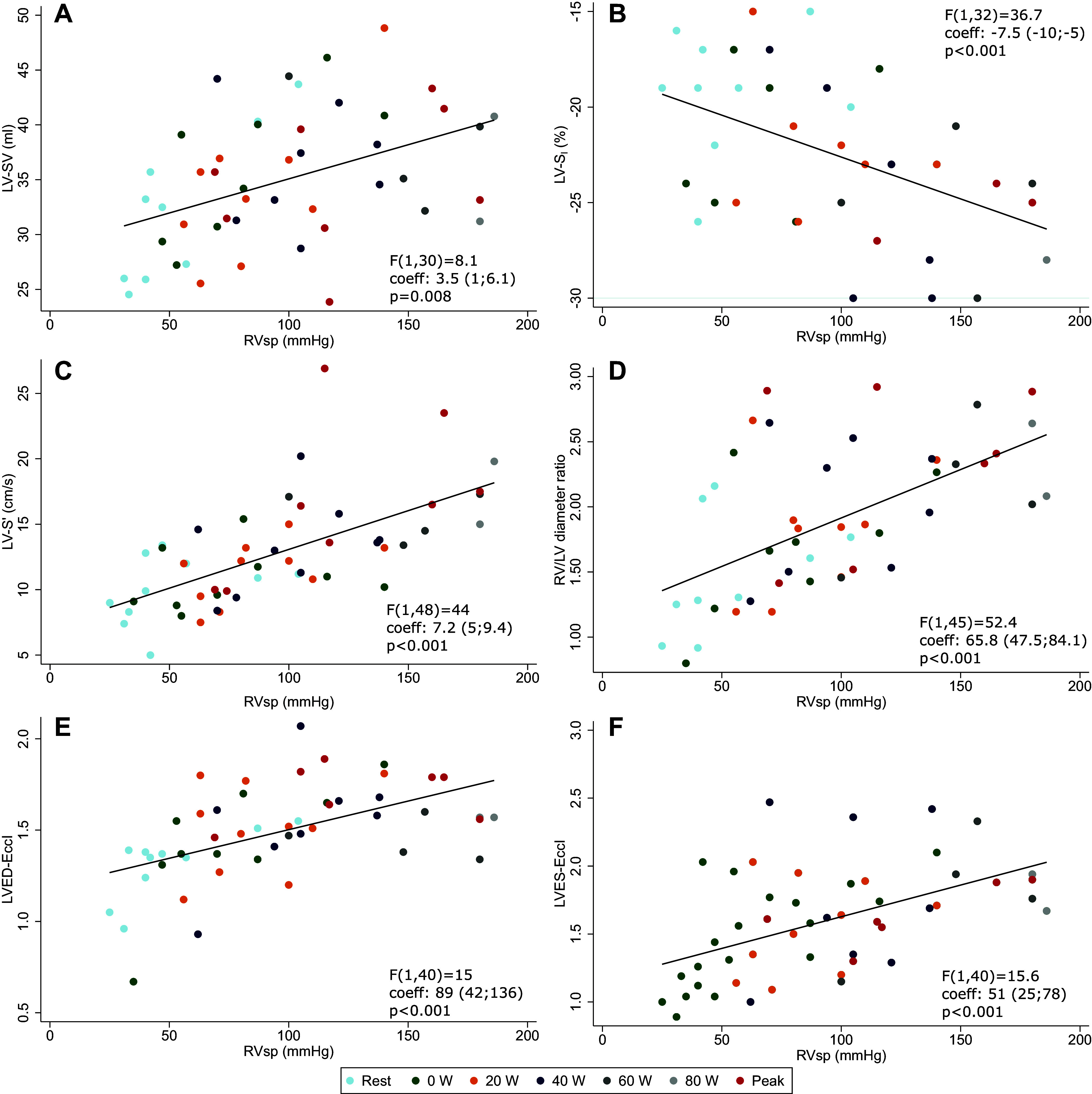
Right ventricle (RV) systolic pressure (RVsp) associations with left ventricular (LV) systolic function and RV-LV interaction parameters. *A*: LV stroke volume (LV-SV). *B*: LV peak systolic longitudinal strain (LV-S_l_). *C*: LV peak longitudinal systolic velocity (RV-S′). *D*: RV:LV diameter ratio. *E*: LV end-diastolic eccentricity index (LVED-EccI). *F*: LV end-systolic eccentricity index (LVES-EccI). *F* values, coefficient, and *P* values are from mixed models with RVsp as dependent variable, cardiac function parameters as independent variable (fixed effect term), and participant cluster as random effect term, not adjusted by exercise intensity step (Table 3 and Supplemental Table S5).

Across the duration of exercise, RVsp was significantly and inversely associated with RV-FAC (Fig. 4*A*) and positively associated with RV-S′ (Fig. 4*B*), RV-E′ (Fig. 4*C*), and RV S:D ratio (Fig. 4*D*). There was a nonstatistically significant inverse association with RVFW-S_l_ (Fig. 4*E*), with fewer data points and substantial spread. TAPSE was not associated with RVsp (Fig. 4*F*). Positive associations were observed between RVsp and RV-LV interaction parameters: LV-SV (Fig. 5*A*), LV-S_l_ (Fig. 5*B*), LV-S′ (Fig. 5*C*), LV-E′ (Fig. 5*D*), LVED-EccI (Fig. 5*E*), and LVES-EccI (Fig. 5*F*). Similar associations were found between HR and the same cardiac parameters (Supplemental Fig. S1 for RV and Supplemental Fig. S2 for LV parameters), likely because of the strong linear relationship between HR and RVsp (Supplemental Fig. S3).

### Relationship Between Metabolic Parameters and Cardiac Function During Exercise

Without adjusting for exercise intensity or heart rate response, of the RV function parameters, only RV-S′ (*P* < 0.001), RV-E′ (*P* < 0.001), RV:LV diameter ratio (*P* < 0.001), and RV S:D ratio (*P* < 0.001) were significantly associated with V̇o_2_. In contrast, measures of RV systolic function including RV-FAC (*P* = 0.9), RVFW-S_l_ (*P* = 0.5), and TAPSE (*P* = 0.1) were not associated with V̇o_2_ independent of exercise intensity (Supplemental Fig. S4). RVsp was significantly associated with V̇o_2_ (*P* < 0.001, Supplemental Fig. S3). Of LV parameters, LV-S_l_ (*P* < 0.001), LV-S′ (*P* < 0.001), and LV-E′ (*P* < 0.001) were associated with V̇o_2_. In contrast, LV-SV (−0.2), LVED-EccI (*P* = 0.1), and LVES-EccI (*P* = 0.1) were not associated with V̇o_2_ independent of exercise intensity (Supplemental Fig. S5).

**Figure 6.**
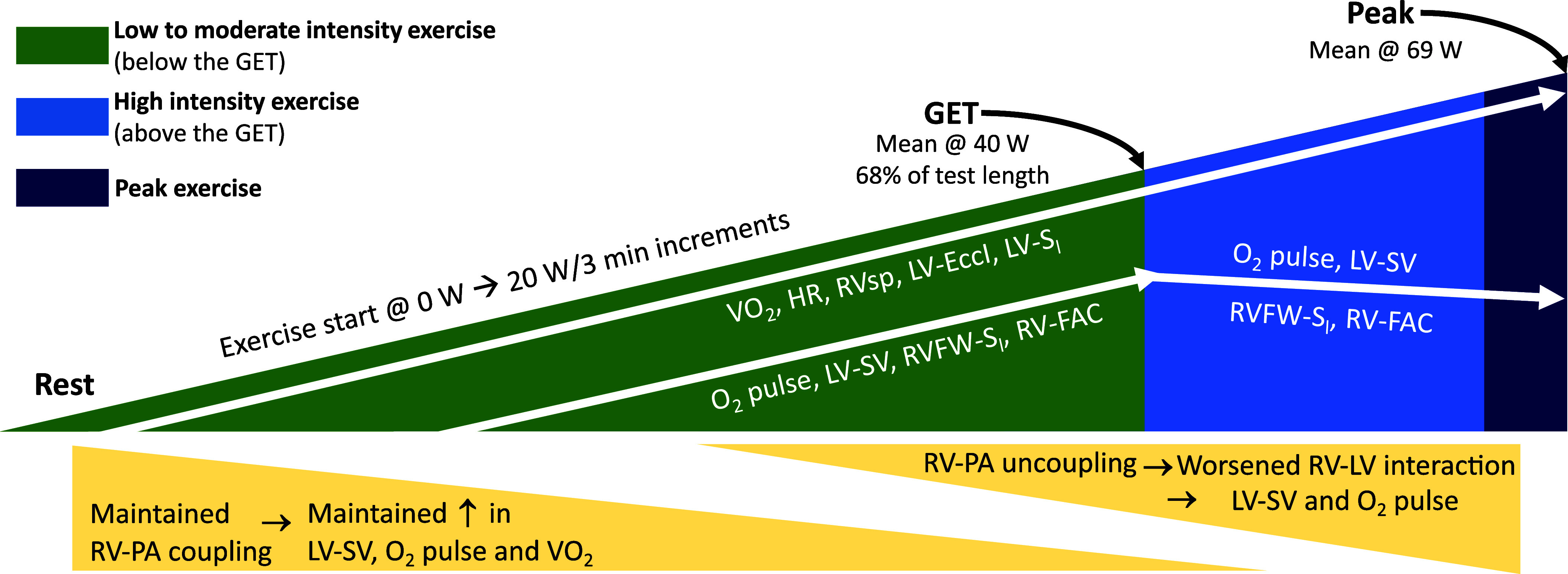
Summary of associations between cardiac function, right ventricle-left ventricle (RV-LV) interaction, and hemodynamic parameters and RV systolic pressure (RVsp), oxygen uptake (V̇o_2_), and O_2_ pulse in relation to the exercise intensity. Numerical data of all associations are presented in Table 3 and Supplemental Tables S5–S8. EccI, eccentricity index; FAC, fractional area change; GET, gas exchange threshold; HR, heart rate; PA, pulmonary artery; RVFW, RV-free wall; S_l_, peak longitudinal strain; SV, stroke volume.

The associations between the cardiac parameters of interest and RVsp, V̇o_2_, and O_2_ pulse differed significantly across the length of the test and are graphically summarized in Fig. 6 and detailed in Table 3 (further numerical data in Supplemental Table S5 and Supplemental Figs. S6–S8). The GET was observed at a mean work rate of 40 ± 8.5 W, with a mean V̇o_2_ of 15.2 mL·min^−1^·kg^−1^, which corresponds to a mean of 69 ± 8.5% of the total test length. This allows for the interpretation of the dynamic relationships between cardiac parameters throughout exercise in relation to exercise intensity. CO was associated with V̇o_2_ independently of exercise intensity, but HR was not. At low- and moderate-intensity exercise, RVsp (and correlated measures, such as RV S:D ratio, RV:LV diameter ratio, and LVES-EccI) were positively associated with V̇o_2_ and O_2_ pulse. The strength of these associations decreased approaching the GET. LV-S_l_ showed a positive association with RVsp, stronger at high intensity and peak exercise, whereas TAPSE showed an inverse relationship with RVsp beyond the GET. RV-FAC and RVFW-S_l_ were positively associated with V̇o_2_ and O_2_ pulse, respectively. This relationship became significant starting with moderate intensity exercise, and the strength of association increased beyond the GET and at peak exercise. LVED-EccI and RV:LV diameter ratios were inversely associated with V̇o_2_ at high intensity and peak exercise, although weakly so.

**Table 3.** Associations between cardiac function parameters and RVsp, V̇o_2_, and O_2_ pulse in relation to increasing exercise intensity

	Main Effect (at 50% of Test Duration)		Interaction Term (with %Test Duration)		Unadjusted by Exercise Intensity	
	Median [CI]	*P* Value	Median [CI]	*P* Value	Median [CI]	*P* Value
	RVsp					
TAPSE, mm	−1.5 [−4;0.1]	0.2	−0.07 [−0.1;−0.02]	0.004 (*P* < 0.1 from 60% of test)	0.4 [−3.9;4.6]	0.9
LV-SV, mL	1.6 [−0.2;3.4]	0.09	−0.02 [−0.4;0.009]	0.2 (*P* < 0.1 up to 50% of test)	3.5 [1;6.1]	0.008
LV FS, %	0.8 [−0.1;1.7]	0.1	0.0002 [−0.02;0.02]	0.9	2.1 [0.8;3.4]	0.002
LVES-EccI	31.94 [7.7;56.1]	<0.01	−0.3 [−0.7;0.07]	0.1 (*P* < 0.1 up to 90% of test)	51.5 [25.2;77.8]	<0.001
RV:LV diameter ratio	34.7 [10.2;59.2]	0.007	−0.07 [−0.4;0.2]	0.6	65.8 [47.5;84.1]	<0.001
LV-S_l_, %	−2.9 [−5.4;−0.4]	0.03	−0.02 [−0.07;0.04]	0.5 (*P* < 0.1 from 10% of test)	−7.5 [−10;−5]	<0.001
	V̇o_2_					
CO, mL·kg^−1^·min^−1^	0.08 [0.05;0.1]	<0.001	0.0008 [0.0003;0.001]	0.003	0.2 [0.2;0.3]	<0.001
RV-FAC, %	0.2 [0.05;0.3]	0.008	0.003 [0.0006;0.005]	0.02 (*P* < 0.1 beyond 30% of test)	−0.02 [−0.4;0.3]	0.9
RV-S_l_, %	−0.07 [−0.3;0.1]	0.5	−0.004 [−0.008;0.0002]	0.06 (*P* < 0.1 beyond 90% of test)	−0.1 [−0.6;0.4]	0.6
LV-SV, mL	−0.1 [−0.2;0.03]	0.1	−0.05 [−0.007;−0.003]	<0.001 (*P* < 0.1 from 50% of test)	−0.2 [−0.5;0.1]	0.2
LV-S_l_, %	−0.2 [−0.4;0.006]	0.06	−0.06 [−0.01;−0.01]	0.008 (*P* < 0.1 from 50% of test)	−0.8 [−1.1;−0.5]	<0.001
LVED-EccI	−1.3 [−5.1;2.5]	0.5	−0.09 [−0.2;−0.03]	0.006 (*P* < 0.01 from 70% of test)	6.3 [−1.4;13.9]	0.1
LVES-EccI	4.1 [1.8;6.3]	0.001	−0.09 [−0.1;−0.06]	<0.001 (*P* < 0.1 up to 80% of test)	3.2 [−0.9;7.4]	0.1
RV:LV diameter ratio	0.3 [−1.9;2.5]	0.8	−0.05 [−0.08;−0.03]	<0.001 (*P* < 0.1 up to 20% and from 80%)	7.7 [4.3;11.2]	<0.001
	O_2_ pulse					
RVFW-S_l_, %	−0.07 [−0.2;0.001]	0.05	−0.0004 [−0.002;0.001]	0.5 (*P* < 0.1 from 30% of test)	−0.03 [−0.2;0.1]	0.7
RVsp, mmHg	0.01 [−0.002;0.03]	0.1	−0.0001 [−0.0003;0.00004]	0.1 (*P* < 0.1 up to 50% of test)	0.03 [0.02;0.04]	<0.001
RV S:D ratio	1.8 [0.2;3.39]	0.03	−0.01 [−0.04;0.02]	0.4 (*P* < 0.1 up to 60%)	4.18 [3.22;5.15]	<0.001
LV-SV, mL	0.1 [0.05;0.2]	0.002	−0.0002 [−0.001;0.0007]	0.6 (*P* < 0.1 throughout the test)	0.2 [0.05;0.2]	0.004
LV FS, %	0.02 [−0.1;0.06]	0.2	−0.001 [−0.002;−0.0003]	0.002 *P* < 0.1 up to 40% of test)	0.06 [0.005;0.1]	0.03
LVES-EccI	1.4 [0.4;2.4]	0.008	−0.2 [−0.04;−0.1]	0.01 (*P* < 0.1 up to 80% of test)	1.4 [0.4;2.4]	0.01
RV:LV diameter ratio	0.7 [−0.4;1.7]	0.2	−0.02 [−0.04;−0.01]	0.001 (*P* < 0.1 up to 50%)	0.8 [−0.06;1.6]	0.07

Values are coefficients [95% confidence intervals (CIs)]. Increasing exercise intensity is defined as percentage from total test duration. EccI, eccentricity index; FAC, fractional area change; FS, fractional shortening; LV, left ventricle; LVED, LV end diastolic; LVES, LV end systolic; RV, right ventricle; RVsp, RV end-systolic pressure; RVFW, RV-free wall; S_l_, peak longitudinal strain; S:D, systolic-to-diastolic ratio; SV, stroke volume; TAPSE, tricuspid annular peak systolic excursion; V̇o_2_, oxygen uptake. All values are from a series of linear mixed models where each cardiac parameter was included as an independent variable with exercise intensity percentage from the total test length as a fixed effect continuous time variable, participant cluster as a random effect, and interaction term between each of the cardiac parameters and time variable. *P* values are shown for main effect (centered around 50% of test length) and coefficients for the continuous-to-continuous interaction term. Main term effect and 95% CI were plotted across the time variable, and time point inflexion where the *P* value became <0.1 was reported, in increments of 10%. Unadjusted models account only for participant cluster random effect. Shown are statistically significant associations, summarized in Fig. 6 Supplemental table S5 details all results from all explored associations. Supplemental Figs. S6–S8 show time-dependent changes in parameter relationships, based on interaction term models.

## DISCUSSION

This study investigated the relationship between biventricular function and cardiopulmonary metabolic parameters during incremental bicycle exercise in children with iPAH using concomitant CPET and echocardiography. Our main results show that *1*) there is a significant increase in RVsp during exercise, accompanied by an increase in RV function during low and moderate exercise; *2*) the ability of the RV functional reserve to meet the increased afterload during exercise determines exercise capacity (RV-PA uncoupling occurred at a similar timing as the GET, at transition from moderate to high-intensity exercise); and *3*) RV-PA uncoupling worsens RV-LV interactions, impacting LV-SV, leading to LV functional reserve contributing to peak exercise capacity, in addition to RV function. These original findings enhance our understanding of the pathophysiology of exercise capacity and limitations in PAH and provide a basis for further research to determine appropriate exercise testing and prescription.

### Normal versus Pathological Cardiac Function Response to Exercise

In healthy children, cardiac function increases during exercise to respond to the additional metabolic requirements (13, 31). This is achieved concurrently by augmenting SV through the Frank–Starling mechanism (32) and contractility to allow for a higher HR by the force-frequency relationship (29). As a result of these physiological mechanisms, systolic cardiac function parameters have been described to increase proportionally to exercise intensity (13, 29, 33, 34). This is regardless of the methodology used, with studies using TDI, STE, or 2-D echocardiography being consistent in describing a similar pattern of steady increase in biventricular systolic function. There has been evidence of a possible plateau in deformation-based indices, such as longitudinal strain, at high-intensity or peak exercise (13, 31, 35), which relates to the plateau in SV that has been described in healthy individuals (32).

Another important characteristic of RV function is the concomitant contribution of multiple components, namely longitudinal, radial and antero-posterior shortening, resulting in the global, three-dimensional systolic function. At rest, the longitudinal component has the most important contribution to global function, but it is not known whether this changes during exercise, with different RV overload states increasing or decreasing the role of other components (36). Moreso, without three-dimensional modalities, each component is assessed separately, and most importantly, each technique has limitations. Thus, it is recommended that the evaluation of the RV systolic function be multiparametric, ideally including TAPSE, RV-S′, RVFW-S_l_, and RV-FAC, and interpreted together (37).

Quantitative assessment of LV and RV diameters and function during incremental exercise in this iPAH cohort revealed several pathophysiological mechanisms that may potentially contribute to the reduced overall exercise capacity. RV function, including RV strain parameters, worsened on average during peak exercise in iPAH children, correlating with reduced O_2_ pulse and a dramatic increase in RVSp. In contrast, cardiac function improves during exercise in healthy children (13, 29), suggesting an important role of impaired myocardial functional reserve in limiting exercise capacity. It is a plausible hypothesis that the normal distensibility of the PA allowing for increased right ventricular cardiac output during exercise is lost in patients with PAH, contributing to markedly increased RVsp.

RV cardiac reserve exercise response was abnormal, resulting from RV-PA uncoupling in the context of RVsp increasing, whereas RV-FAC and RVFW-S_l_ decreased. Different patterns were observed with different aspects of RV function, for example, RV-S′ increased through exercise, whereas RVFW-S_l_ decreased. We speculate that this reflects inherent mechanistic processes, with velocity-based measures such as RV-S′ being related to heart rate and the force-frequency relation, whereas deformation-based measures such as RVFW-S_l_ and RV-FAC are more related to volume changes and the Frank–Starling mechanism. It is worth noting that the modality of acquisition and estimation is different for each, with different sources of error during exercise, such as breathing movement, frame-rate limitation, and Doppler insonation angle, all impacting the results. The increases in RVsp were associated with an increased RV S:D ratio and reduced LV preload, as observed by the worsening LV-EccI. In addition, there was an inability to augment stroke volume despite the present, although reduced, LV functional reserve. This shows that the impaired RV exercise response may be driven by insufficient RV contractile reserve to satisfy the simultaneous demands of exercise and the resulting pathological increase in PA pressure together with adverse RV-LV interactions. This leads to RV-PA uncoupling and an inability to maintain LV-SV during moderate-intensity exercise due to increased LV EccI (38). Beyond moderate-exercise intensity (i.e., beyond the GET), RV dysfunction becomes associated with exercise capacity. Closer to peak exercise, LV systolic dysfunction and abnormal RV-LV interaction become inversely associated with V̇o_2_ and O_2_ pulse. Ultimately, these cascading effects might have contributed to the significantly reduced peak oxygen consumption when compared with values reported in similar age healthy participants (13).

In our cohort, there was evidence of a pathologically altered RV force-frequency relationship (FFR) during exercise (39), approximated by an inverse relation between RV-FAC/RVFW-S_l_ and HR. This is the opposite of the positive FFR described during exercise in healthy children (13, 40). Moreso, an inverse relation between RV-FAC/RVFW-S_l_ and RVsp indicates impaired RV-PA coupling during exercise. In contrast, TDI parameters maintained a positive association with both HR and RVsp, possibly also due to increasing translational cardiac motion during exercise, which affects TDI measurements. These data emphasize the need to measure both global and longitudinal components of RV function, shown to be correlated to oxygen uptake (16), rather than relying on one single parameter.

### Importance of RV-PA Coupling and RV-LV Interaction during Exercise

In healthy children and adolescents, pulmonary distensibility and RV-PA coupling were described to be superior to adults at exercise, highlighting the increased ability to adapt to the augmentation of cardiac output without a disproportionate rise in RVsp (41). Markers of exercise RV-PA coupling, such as the cardiac output to PA pressure slope were recently described in detail in a large adult cohort of mixed right heart diseases, with an important group of healthy controls, some of them athletes, which showed high sensitivity in identifying pathology (42). From a physiological perspective, RV-PA coupling control is crucial in maintaining optimal systolic efficiency, with an invasive ventricular-to-pulmonary arterial elastance ratio being maintained within narrow ranges, even with impressive changes in cardiac output seen in exercise (43). A less-studied phenomenon during exercise, with no work published to that that we are aware of, is the downstream RV to LV interaction during exercise due to physiological increases in RVsp. Most studies measuring SV at exercise describe a continuous increase, or at most a plateau at peak, with very few describing a decrease, attributed to detraining status or older age. Thus, it is plausible that unfavorable RV-LV interactions do not occur to any significant extent in healthy children during exercise. It is thus reasonable to assume that the physiological increase in RVsp in healthy children is not significant enough (44) to adversely impact normal LV function.

In our cohort, RVSp during exercise rose beyond the ∼50 mmHg reported in healthy adults during exercise (44). The ability to increase RVSp during exercise in patients with PAH can indicate RV contractile reserve, which in turn is associated with better outcomes (16), although the high PVRi in PAH requires high RV contractile forces during exercise to maintain adequate pulmonary flow (45). In the current study, RVsp increased steadily during exercise, following the increase in HR. RVsp was positively associated with O_2_ pulse for the initial part of the exercise, as was LVES-EccI. At higher exercise intensities, RV dysfunction was associated with worse oxygen uptake and O_2_ pulse, congruent with the abnormal RV-PA coupling discussed earlier. The detrimental impact of high RVSp on LV compression and filling is expected to reduce exercise capacity (20, 38). This leads to reduced stroke volume and exercise tolerance as we observed in the current study, although without investigating maximal LV-EccI (38). These LV-RV interactions determine ventricular filling and cardiac output during exercise. At high intensity, lower LV-S_l_ was strongly associated with lower concomitant V̇o_2_. At peak exercise, LVED-EccI was inversely associated with V̇o_2_ emphasizing the role of LV-RV mechanical interaction in exercise limitations in this population (19). Impaired diastolic coronary artery perfusion resulting from prolonged systolic duration may further contribute to this pathophysiology and requires further investigation.

### Clinical Implications and Future Directions

In current clinical practice, exercise echocardiography and, to some degree, CPET remain underused diagnostic and prognostic tools (46, 47), possibly more so in children with PAH. These tests involve high-intensity or maximal exercise, which might be perceived as difficult or even risky in these patients. Furthermore, acquiring good-quality images has been considered difficult, limiting the use of these modalities (48). Current European guidelines emphasize the role of CPET in the diagnosis and follow-up of PAH, but state exercise stress echocardiography is yet to have an established use (5). In our cohort, all participants were able to exercise beyond their GET, into the high-intensity domain, without adverse events. Nonetheless, the issue of image quality remains relevant, especially for STE at peak, and it is important that testing protocols be optimized.

When exercise stress echocardiography is performed, there is little to no guidance on what parameters are prognostic and associated with the severity of the disease. Recent evidence has shown the superior prognostic value of RV-PA coupling over other imaging parameters at rest (18). Thus, it is reasonable to study the RV-PA unit during exercise. In this study, we show that it is the RV-PA coupling and worsening RV-LV interactions that correlate with exercise capacity and limitation. Furthermore, our results emphasize the importance of high-intensity exercise testing, as assessments below the GET are likely to miss the full pathological response, which we show occurs beyond this point. Using a combined CPET and echocardiography approach provides even more information, as cardiac output to PA pressure slopes can be determined, as shown recently in a large study of right heart disease (42).

Thus, future work should focus on improving protocols for combined CPET and echocardiography exercise testing to allow for more personalized and streamlined testing in complex populations such as PAH. Our group has recently developed a single-stage high-intensity exercise testing protocol, which takes into consideration the GET as an individual fitness threshold (49). A simplified protocol such as this, potentially including a moderate intensity step, could be more easily implemented in clinical practice and allow for longitudinal evaluation to determine whether changes in RV-PA coupling and RV-LV interaction precede a decline in cardiorespiratory fitness. These could also potentially be useful surrogate endpoints in therapeutic clinical trials, beyond survival and subjective clinical worsening. Finally, by identifying the relationship between cardiac reserve and exercise intensity, more personalized physical rehabilitation could potentially be prescribed, guided not just by symptoms but also by pinpointing the underlying pathological mechanisms that limit exercise.

### Limitations

A limitation of this study is the small sample size, despite the rare disease categorization and the pediatric population. Consequently, multivariable analysis of factors associated with cardiopulmonary response to exercise was not attempted outside of adjusting for exercise intensity. As such, some associations could be mediated through confounding relationships, rather than direct, and this was accounted for in how the findings were interpreted in the physiological context. Because of the small sample size, the confidence intervals of some estimates were wide and were interpreted with caution when this was the case. During exercise testing, the quality of the images obtained is variable, as previously documented (29, 39), leading to data gaps, which can introduce bias, even if gaps are assumed as random. To allow for the interpretation of results in terms of exercise intensity domains, these were approximated by standardizing according to each individual’s GET and its relative average in relation to the peak test values. Despite limitations on how accurately GET can be assessed, in a protocol with fixed increments, this still allows for more physiological comparisons than based on work rate or heart rate alone. Estimated RVsp was used in the analysis, and this has some limitations when approximating true pulmonary systolic pressure. Some of the functional parameters evaluated, such as the S:D ratio, are linked to heart rate, which changes with exercise. In preliminary analysis, we determined that none of the currently used methods for heart rate adjustments of the S:D ratio is usable during exercise without extreme bias at high intensity. Instead, we relied on the fact that this is a ratio measure, which will have a constant “bias” at each exercise intensity level, impacted by the heart rate at each intensity. Therefore, we adjusted for this more physiological parameter instead. Whether this overcomes the inherent issues of analyzing timing data at extreme heart rates is still to be determined. Finally, the recruited population is limited to those able to exercise, and thus might not reflect the subset of patients that are in worse functional status.

### Conclusions

In children with PAH, it would appear that it is not the increase in pulmonary pressure alone that limits peak exercise, but rather the concomitant reduced RV functional reserve. Thus, assessment of RV function during exercise, in addition to assessment of PA pressures, may be important for clinical assessment of patients with PAH. These combined responses result in RV-PA uncoupling at high-intensity exercise, subsequent worsening of interventricular interaction, and LV dysfunction. Overall, our results inform the need for, and implementation of, functional exercise testing in this population for better cardiorespiratory fitness characterization, possible prognostic stratification, and exercise prescription.

## DATA AVAILABILITY

Deidentified data used in this analysis cannot be made publicly available as it might contain sensitive information but can be shared upon reasonable request to the corresponding author, and data sharing agreements compliant with local and international data privacy laws are implemented.

## SUPPLEMENTAL MATERIAL

10.6084/m9.figshare.26018326.v1 Supplemental Figs. S1–S8 and Supplemental Tables S1–S5: https://doi.org/10.6084/m9.figshare.26018326.v1.

## GRANTS

G.E. Pieles was supported through the Susan Harris and David Kassie Fellowship in Cardiology from the Labatt Family Heart Center (2014–2016). D.M. Dorobantu is supported by a doctoral scholarship (Grant MR/N0137941/1 for the GW4 BIOMED DTP, awarded to the Universities of Bath, Bristol, Cardiff and Exeter from the Medical Research Council (MRC)/UKRI (2019–2023).

## DISCLOSURES

No conflicts of interest, financial or otherwise, are declared by the authors.

## AUTHOR CONTRIBUTIONS

G.E.P., D.-M.D., J.E.C., B.C., J.R., S.R.R., E.H., T.H., L.M., G.D.W., and M.K.F. conceived and designed research; G.E.P., D.-M.D., J.E.C., B.C., J.R., E.H., and C.A.W. performed experiments; G.E.P., D.-M.D., J.E.C., B.C., E.H., and L.M. analyzed data; G.E.P., D.-M.D., J.E.C., B.C., J.R., S.R.R., E.H., C.A.W., T.H., L.M., G.D.W., and M.K.F. interpreted results of experiments; G.E.P., D.-M.D., and E.H. prepared figures; G.E.P., D.-M.D., B.C., S.R.R., E.H., C.A.W., T.H., L.M., G.D.W., and M.K.F. drafted manuscript; G.E.P., D.-M.D., J.E.C., B.C., J.R., S.R.R., E.H., C.A.W., T.H., L.M., G.D.W., and M.K.F. edited and revised manuscript; G.E.P., D.-M.D., J.E.C., B.C., J.R., S.R.R., E.H., C.A.W., T.H., L.M., G.D.W., and M.K.F. approved final version of manuscript.

## References

[1] Humbert M, Sitbon O, Chaouat A, Bertocchi M, Habib G, Gressin V, Yaici A, Weitzenblum E, Cordier JF, Chabot F, Dromer C, Pison C, Reynaud-Gaubert M, Haloun A, Laurent M, Hachulla E, Simonneau G. Pulmonary arterial hypertension in France: results from a national registry. Am J Respir Crit Care Med 173: 1023–1030, 2006. doi:10.1164/rccm.200510-1668OC. 16456139

[2] Duffels MG, Engelfriet PM, Berger RM, van Loon RL, Hoendermis E, Vriend JW, van der Velde ET, Bresser P, Mulder BJ. Pulmonary arterial hypertension in congenital heart disease: an epidemiologic perspective from a Dutch registry. Int J Cardiol 120: 198–204, 2007. doi:10.1016/j.ijcard.2006.09.017. 17182132

[3] Miyamoto S, Nagaya N, Satoh T, Kyotani S, Sakamaki F, Fujita M, Nakanishi N, Miyatake K. Clinical correlates and prognostic significance of six-minute walk test in patients with primary pulmonary hypertension: comparison with cardiopulmonary exercise testing. Am J Respir Crit Care Med 161: 487–492, 2000. doi:10.1164/ajrccm.161.2.9906015. 10673190

[4] McLaughlin VV, Archer SL, Badesch DB, Barst RJ, Farber HW, Lindner JR, , et al A report of the American College of Cardiology foundation task force on expert consensus documents and the American Heart Association. Circulation 119: 2250–2294, 2009 [Erratum in *Circulation* 120: e13, 2009]. doi:10.1161/CIRCULATIONAHA.109.192230. 19332472

[5] Humbert M, Kovacs G, Hoeper MM, Badagliacca R, Berger RMF, Brida M, Carlsen J, Coats AJS, Escribano-Subias P, Ferrari P, Ferreira DS, Ghofrani HA, Giannakoulas G, Kiely DG, Mayer E, Meszaros G, Nagavci B, Olsson KM, Pepke-Zaba J, Quint JK, Rådegran G, Simonneau G, Sitbon O, Tonia T, Toshner M, Vachiery JL, Vonk Noordegraaf A, Delcroix M, Rosenkranz S; ESC/ERS Scientific Document Group. 2022 ESC/ERS Guidelines for the diagnosis and treatment of pulmonary hypertension. Eur Heart J 43: 3618–3731, 2022 [Erratum in *Eur Heart J* 44: 1312, 2023]. doi:10.1093/eurheartj/ehac237. 36017548

[6] Sun XG, Hansen JE, Oudiz RJ, Wasserman K. Exercise pathophysiology in patients with primary pulmonary hypertension. Circulation 104: 429–435, 2001. doi:10.1161/hc2901.093198. 11468205

[7] Wensel R, Opitz CF, Anker SD, Winkler J, Höffken G, Kleber FX, Sharma R, Hummel M, Hetzer R, Ewert R. Assessment of survival in patients with primary pulmonary hypertension: importance of cardiopulmonary exercise testing. Circulation 106: 319–324, 2002. doi:10.1161/01.CIR.0000022687.18568.2A. 12119247

[8] Deboeck G, Scoditti C, Huez S, Vachiéry JL, Lamotte M, Sharples L, Melot C, Naeije R. Exercise testing to predict outcome in idiopathic versus associated pulmonary arterial hypertension. Eur Respir J 40: 1410–1419, 2012. doi:10.1183/09031936.00217911. 22441747

[9] Yetman AT, Taylor AL, Doran A, Ivy DD. Utility of cardiopulmonary stress testing in assessing disease severity in children with pulmonary arterial hypertension. Am J Cardiol 95: 697–699, 2005. doi:10.1016/j.amjcard.2004.10.056. 15721127

[10] Cheng S, Li VWY, Cheung YF. Systolic and diastolic functional reserve of the subpulmonary and systemic right ventricles as assessed by pharmacologic and exercise stress: a systematic review. Echocardiography 39: 310–329, 2022. doi:10.1111/echo.15285. 34997638

[11] Rosenzweig EB, Abman SH, Adatia I, Beghetti M, Bonnet D, Haworth S, Ivy DD, Berger RMF. Paediatric pulmonary arterial hypertension: updates on definition, classification, diagnostics and management. Eur Respir J 53: 1801916, 2019. doi:10.1183/13993003.01916-2018. 30545978 PMC6351335

[12] Rowland T. Echocardiography and circulatory response to progressive endurance exercise. Sports Med 38: 541–551, 2008. doi:10.2165/00007256-200838070-00002. 18557657

[13] Pieles GE, Gowing L, Forsey J, Ramanujam P, Miller F, Stuart AG, Williams CA. The relationship between biventricular myocardial performance and metabolic parameters during incremental exercise and recovery in healthy adolescents. Am J Physiol Heart Circ Physiol 309: H2067–H2076, 2015. doi:10.1152/ajpheart.00627.2015. 26475589 PMC4698429

[14] Cote AT, Duff DK, Escudero CA, De Souza AM, Williams LD, Gill R, Zadorsky MT, Harris KC, Potts JE, Sandor GGS. A Doppler echocardiographic study of the myocardial inotropic response to peak semisupine exercise in healthy children: development of a simplified index of myocardial reserve. J Am Soc Echocardiogr 30: 790–796, 2017. doi:10.1016/j.echo.2017.04.008. 28599828

[15] Cifra B, Dragulescu A, Border WL, Mertens L. Stress echocardiography in paediatric cardiology. Eur Heart J Cardiovasc Imaging 16: 1051–1059, 2015. doi:10.1093/ehjci/jev159. 26130262

[16] Grünig E, Tiede H, Enyimayew EO, Ehlken N, Seyfarth HJ, Bossone E, D’Andrea A, Naeije R, Olschewski H, Ulrich S, Nagel C, Halank M, Fischer C. Assessment and prognostic relevance of right ventricular contractile reserve in patients with severe pulmonary hypertension. Circulation 128: 2005–2015, 2013. doi:10.1161/CIRCULATIONAHA.113.001573. 24056689

[17] Hansmann G, Koestenberger M, Alastalo TP, Apitz C, Austin ED, Bonnet D, Budts W, D’Alto M, Gatzoulis MA, Hasan BS, Kozlik-Feldmann R, Kumar RK, Lammers AE, Latus H, Michel-Behnke I, Miera O, Morrell NW, Pieles G, Quandt D, Sallmon H, Schranz D, Tran-Lundmark K, Tulloh RMR, Warnecke G, Wåhlander H, Weber SC, Zartner P. 2019 updated consensus statement on the diagnosis and treatment of pediatric pulmonary hypertension: the European Pediatric Pulmonary Vascular Disease Network (EPPVDN), endorsed by AEPC, ESPR and ISHLT. J Heart Lung Transplant 38: 879–901, 2019. doi:10.1016/j.healun.2019.06.022. 31495407

[18] Ünlü S, Bézy S, Cvijic M, Duchenne J, Delcroix M, Voigt JU. Right ventricular strain related to pulmonary artery pressure predicts clinical outcome in patients with pulmonary arterial hypertension. Eur Heart J Cardiovasc Imaging 24: 635–642, 2023. doi:10.1093/ehjci/jeac136. 35852912

[19] Gan C, Lankhaar JW, Marcus JT, Westerhof N, Marques KM, Bronzwaer JG, Boonstra A, Postmus PE, Vonk-Noordegraaf A. Impaired left ventricular filling due to right-to-left ventricular interaction in patients with pulmonary arterial hypertension. Am J Physiol Heart Circ Physiol 290: H1528–H1533, 2006. doi:10.1152/ajpheart.01031.2005. 16284226

[20] Burkett DA, Slorach C, Patel SS, Redington AN, Ivy DD, Mertens L, Younoszai AK, Friedberg MK. Impact of pulmonary hemodynamics and ventricular interdependence on left ventricular diastolic function in children with pulmonary hypertension. Circ Cardiovasc Imaging 9: e004612, 2016. doi:10.1161/CIRCIMAGING.116.004612. 27581953 PMC5012318

[21] Galiè N, Humbert M, Vachiery JL, Gibbs S, Lang I, Torbicki A, Simonneau G, Peacock A, Noordegraaf AV, Beghetti M, Ghofrani A, Sanchez MA, Hansmann G, Klepetko W, Lancellotti P, Matucci M, McDonagh T, Pierard LA, Trindade PT, Zompatori M, Hoeper M. 2015 ESC/ERS Guidelines for the diagnosis and treatment of pulmonary hypertension. Eur Respir J 46: 903–975, 2015. doi:10.1183/13993003.01032-2015. 26318161

[22] Ferreira LF, Lutjemeier BJ, Townsend DK, Barstow TJ. Dynamics of skeletal muscle oxygenation during sequential bouts of moderate exercise. Exp Physiol 90: 393–401, 2005. doi:10.1113/expphysiol.2004.029595. 15708875

[23] Poole DC, Rossiter HB, Brooks GA, Gladden LB. The anaerobic threshold: 50+ years of controversy. J Physiol 599: 737–767, 2021. doi:10.1113/JP279963. 33112439

[24] Beaver WL, Wasserman K, Whipp BJ. A new method for detecting anaerobic threshold by gas exchange. J Appl Physiol (1985) 60: 2020–2027, 1986. doi:10.1152/jappl.1986.60.6.2020. 3087938

[25] Lopez L, Colan SD, Frommelt PC, Ensing GJ, Kendall K, Younoszai AK, Lai WW, Geva T. Recommendations for quantification methods during the performance of a pediatric echocardiogram: a report from the pediatric measurements writing group of the American Society of Echocardiography Pediatric and Congenital Heart Disease Council. J Am Soc Echocardiogr 23: 465–495, 2010. doi:10.1016/j.echo.2010.03.019. 20451803

[26] Jone PN, Hinzman J, Wagner BD, Ivy DD, Younoszai A. Right ventricular to left ventricular diameter ratio at end-systole in evaluating outcomes in children with pulmonary hypertension. J Am Soc Echocardiogr 27: 172–178, 2014. doi:10.1016/j.echo.2013.10.014. 24325962 PMC3922965

[27] Alkon J, Humpl T, Manlhiot C, McCrindle BW, Reyes JT, Friedberg MK. Usefulness of the right ventricular systolic to diastolic duration ratio to predict functional capacity and survival in children with pulmonary arterial hypertension. Am J Cardiol 106: 430–436, 2010. doi:10.1016/j.amjcard.2010.03.048. 20643259

[28] Friedberg MK. Imaging right-left ventricular interactions. JACC Cardiovasc Imaging 11: 755–771, 2018. doi:10.1016/j.jcmg.2018.01.028. 29747850

[29] Cifra B, Mertens L, Mirkhani M, Slorach C, Hui W, Manlhiot C, Friedberg MK, Dragulescu A. Systolic and diastolic myocardial response to exercise in a healthy pediatric cohort. J Am Soc Echocardiogr 29: 648–654, 2016. doi:10.1016/j.echo.2016.02.015. 27038512

[30] Cifra B, Chen CK, Fan CPS, Slorach C, Manlhiot C, McCrindle BW, Dragulescu A, Redington AN, Friedberg MK, Nathan PC, Mertens L. Dynamic myocardial response to exercise in childhood cancer survivors treated with anthracyclines. J Am Soc Echocardiogr 31: 933–942, 2018. doi:10.1016/j.echo.2018.02.003. 29615292

[31] Dorobantu D, Wadey CA, Ryding D, Mcnally S, Perry D, Friedberg M, Stuart AG, Pieles GE, Williams CA. Relationships between biventricular myocardial function and oxygen uptake during exercise in healthy adolescent male athletes. Eur Heart J Cardiovasc Imaging 24: jead119–319, 2023. doi:10.1093/ehjci/jead119.319.

[32] Vieira SS, Lemes B, de T C de Carvalho P, N de Lima R, S Bocalini D, A S Junior J, Arsa G, A Casarin C, L Andrade E, J Serra A. Does stroke volume increase during an incremental exercise? A systematic review. Open Cardiovasc Med J 10: 57–63, 2016. doi:10.2174/1874192401610010057. 27347221 PMC4896996

[33] Rowland TW, Garrard M, Marwood S, Guerra ME, Roche D, Unnithan VB. Myocardial performance during progressive exercise in athletic adolescent males. Med Sci Sports Exerc 41: 1721–1728, 2009. doi:10.1249/MSS.0b013e3181a06cb5. 19657299

[34] von Scheidt F, Kiesler V, Kaestner M, Bride P, Krämer J, Apitz C. Left ventricular strain and strain rate during submaximal semisupine bicycle exercise stress echocardiography in healthy adolescents and young adults: systematic protocol and reference values. J Am Soc Echocardiogr 33: 848–857.e1, 2020. doi:10.1016/j.echo.2019.12.015. 32122743

[35] Pieles GE, Gowing L, Ryding D, Perry D, McNally SR, Stuart AG, Williams CA. Characterisation of LV myocardial exercise function by 2-D strain deformation imaging in elite adolescent footballers. Eur J Appl Physiol 121: 239–250, 2021. doi:10.1007/s00421-020-04510-6. 33030575 PMC7815563

[36] Kovács A, Lakatos B, Tokodi M, Merkely B. Right ventricular mechanical pattern in health and disease: beyond longitudinal shortening. Heart Fail Rev 24: 511–520, 2019. doi:10.1007/S10741-019-09778-1/TABLES/1. 30852772 PMC6559995

[37] Badano LP, Addetia K, Pontone G, Torlasco C, Lang RM, Parati G, Muraru D. Advanced imaging of right ventricular anatomy and function. Heart 106: 1469–1476, 2020. doi:10.1136/heartjnl-2019-315178. 32620556

[38] Burkett DA, Patel SS, Mertens L, Friedberg MK, Ivy DD. Relationship between left ventricular geometry and invasive hemodynamics in pediatric pulmonary hypertension. Circ Cardiovasc Imaging 13: e009825, 2020. doi:10.1161/CIRCIMAGING.119.009825. 32408829 PMC7236425

[39] Roche SL, Grosse-Wortmann L, Friedberg MK, Redington AN, Stephens D, Kantor PF. Exercise echocardiography demonstrates biventricular systolic dysfunction and reveals decreased left ventricular contractile reserve in children after tetralogy of Fallot repair. J Am Soc Echocardiogr 28: 294–301, 2015. doi:10.1016/j.echo.2014.10.008. 25459500

[40] Roche SL, Vogel M, Pitkänen O, Grant B, Slorach C, Fackoury C, Stephens D, Smallhorn J, Benson LN, Kantor PF, Redington AN. Isovolumic acceleration at rest and during exercise in children: normal values for the left ventricle and first noninvasive demonstration of exercise-induced force-frequency relationships. J Am Coll Cardiol 57: 1100–1107, 2011. doi:10.1016/j.jacc.2010.09.063. 21349402

[41] Forton K, Motoji Y, Caravita S, Faoro V, Naeije R. Exercise stress echocardiography of the pulmonary circulation and right ventricular-arterial coupling in healthy adolescents. Eur Heart J Cardiovasc Imaging 22: 688–694, 2021. doi:10.1093/ehjci/jeaa085. 32380528

[42] Gargani L, Pugliese NR, De Biase ND, Mazzola M, Agoston G, Arcopinto M, , et al Exercise stress echocardiography of the right ventricle and pulmonary circulation. J Am Coll Cardiol 82: 1973–1985, 2023. doi:10.1016/j.jacc.2023.09.807. 37968015

[43] Chantler PD, Lakatta EG, Najjar SS. Arterial-ventricular coupling: mechanistic insights into cardiovascular performance at rest and during exercise. J Appl Physiol (1985) 105: 1342–1351, 2008 [Erratum in *J Appl Physiol* 106:1027, 2009]. doi:10.1152/japplphysiol.90600.2008. 18617626 PMC2576043

[44] Argiento P, Vanderpool RR, Mulè M, Russo MG, D’Alto M, Bossone E, Chesler NC, Naeije R. Exercise stress echocardiography of the pulmonary circulation: limits of normal and sex differences. Chest 142: 1158–1165, 2012. doi:10.1378/chest.12-0071. 22539647 PMC3494470

[45] Janicki JS, Weber KT, Likoff MJ, Fishman AP. The pressure-flow response of the pulmonary circulation in patients with heart failure and pulmonary vascular disease. Circulation 72: 1270–1278, 1985. doi:10.1161/01.CIR.72.6.1270. 4064271

[46] Del Punta L, De Biase N, Armenia S, Di Fiore V, Maremmani D, Gargani L, Mazzola M, De Carlo M, Mengozzi A, Lomonaco T, Galeotti GG, Dini FL, Masi S, Pugliese NR. Combining cardiopulmonary exercise testing with echocardiography: a multiparametric approach to the cardiovascular and cardiopulmonary systems. Eur Heart J Imaging Methods Pract 1: 12, 2023. doi:10.1093/ehjimp/qyad021. PMC11195726 39044798

[47] Reeves T, Bates S, Sharp T, Richardson K, Bali S, Plumb J, Anderson H, Prentis J, Swart M, Levett DZH; Perioperative Exercise Testing and Training Society (POETTS). Cardiopulmonary exercise testing (CPET) in the United Kingdom—a national survey of the structure, conduct, interpretation and funding. Perioper Med (Lond) 7: 2–8, 2018 [Erratum in Perioper Med (Lond) 7: 8, 2018]. doi:10.1186/s13741-017-0082-3. 29423173 PMC5787286

[48] Argiento P, Chesler N, Mulè M, D’Alto M, Bossone E, Unger P, Naeije R. Exercise stress echocardiography for the study of the pulmonary circulation. Eur Respir J 35: 1273–1278, 2010. doi:10.1183/09031936.00076009. 19926746 PMC2879460

[49] Dorobantu DM, Wadey CA, Berryman B, Amir NH, Forsythe L, Stuart AG, Pieles GE, Williams CA. A new protocol for a single-stage combined cardiopulmonary and echocardiography exercise test: a pilot study. Eur Heart J Imaging Methods Pract 2: 21, 2024. doi:10.1093/ehjimp/qyae021. 39045209 PMC11195695

